# Systematic approach to musculoskeletal benign tumors

**DOI:** 10.1097/IJ9.0000000000000046

**Published:** 2017-11-14

**Authors:** Masood Umer, Obada H.A. Hasan, Dawar Khan, Nasir Uddin, Shahryar Noordin

**Affiliations:** The Aga Khan University Hospital (AKUH), Karachi 74800, Pakistan

**Keywords:** Musculoskeletal, Tumors, Radiology

## Abstract

The radiologic workup of musculoskeletal tumors can be both cost-efficient and extremely helpful to the referring clinician if one proceeds in a thoughtful and logical manner. It should be remembered that plain films remain the most reliable imaging method for assessment of both biological activity and probable histologic diagnosis of an osseous lesion. Further investigations are of help to determine the extent of lesion and to help in staging. In order to do this, we have found it useful to include an assessment of 10 determinants in the description of a tumor. If these determinants are accurately described, the correct diagnosis or at least a limited differential diagnosis usually becomes obvious.

The radiologic workup of musculoskeletal tumors can be both cost-efficient and extremely helpful to the referring clinician if one proceeds in a thoughtful and logical manner^1^.

Initially, a musculoskeletal tumor should be simply imaged with a plain film. It should be remembered that plain films remain the most reliable imaging method for assessment of both biological activity and probable histologic diagnosis of an osseous lesion^2^. Although soft tissue involvement by an osseous lesion may be incompletely assessed by plain film, the osseous findings are seen with much better resolution on plain radiographs than with either computed tomography or magnetic resonance^3^. Plain film therefore is used to arrive at a reasonable differential diagnosis or at least to categorize the lesion as to degree of aggressiveness. In order to do this, we have found it useful to include an assessment of 10 determinants in the description of a tumor. If these determinants are accurately described, the correct diagnosis or at least a limited differential diagnosis usually becomes obvious. These determinants are as follows:

1. Age of the patient. This can be an extremely important determinant in some lesions in which the age range of occurrence may be quite narrow^4^. For example, malignant osseous lesions in patients under 1 year of age are usually metastatic neuroblastoma. Malignant osseous lesions in the age range of 1–30 are usually osteosarcoma or Ewing sarcoma. Malignant osseous lesions in the 30- to 60-year range most commonly will be either chondrosarcoma, primary lymphoma, or malignant fibrous histiocytoma, while malignant lesions in the age range over 50 most commonly will be due to metastatic disease or multiple myeloma. Several other osseous lesions have fairly limited age ranges as well. These will be discussed with the individual cases later on in the section.
2. Soft tissue involvement. Cortical breakthrough of a bone lesion to create a soft tissue mass generally suggests an aggressive lesion^5^,^6^. Such soft tissue masses will often distort but not obliterate nearby muscle planes (**Fig. 1**).
3. Pattern of bone destruction^4^. Common terminology includes the terms “geographic” (**Figs. 2, 3**) (well-defined or map-like lesion, the least aggressive pattern), “moth-eaten” (holes, with less well-defined margins, appearing more aggressive), and “permeative” (**Fig. 4**) (a poorly demarcated pattern which is often very difficult to visualize and represents a highly aggressive lesion). It is not always easy to differentiate between the moth-eaten and permeative patterns. Furthermore, since both represent an aggressive pattern, it is not necessary to differentiate between the 2, and the term permeative should serve well for both^7^.
4. Size of lesion. Generally, a larger lesion (>5 cm) is more likely to be malignant or aggressive^8^, but there are many exceptions to this statement, and other determinants are generally more important than this one.
5. Location of the lesion. Three different types of locations should be noted: (**Fig. 5**) the particular bone that is involved, the location in a transverse axis, and the location in a longitudinal axis of a long bone^9^. Occasionally the particular bone involved may be important to the diagnosis. One such example is the tibia, which in addition to hosting most tumors that one can think of, also is the most common location for 3 uncommon tumors, adamantinoma^10^, ossifying fibroma, and chondromyxoid fibroma^11^. Other categorizations of particular bone involvement might be useful such as axial versus appendicular or flat versus tubular bones, with many lesions clearly favoring one over the other^12^,^13^. It is worthwhile to categorize a lesion’s location in the transverse axis of a tubular bone (central, eccentric, or a cortically based epicenter)^14^. As will be noted in discussions of individual tumors later on, many tumors have very characteristic locations in the transverse axis. Similarly, many tumors have characteristic locations along the long axis of a tubular bone (epiphysis, metaphysis, or diaphysis)^7^,^14^. Therefore, this location should be identified in the description of the osseous lesion as well.
6. Zone of transition of the lesion from abnormal to normal bone (**Figs. 6, 7**). A wide zone of transition denotes an aggressive lesion, while a narrow zone is a much less aggressive lesion^15^.
7. Margination of the lesion (**Figs. 8, 9**). A sclerotic margin generally represents a nonaggressive lesion, whereas a nonsclerotic margin often represents an aggressive lesion. There are, however, important exceptions to this, including giant cell tumor and enchondroma. It is generally true that the determinants narrow zone of transition and sclerotic margin occur together in a lesion and suggest that it is nonaggressive^4^. However, these terms are not synonymous. Similarly, the determinants wide zone of transition and nonsclerotic margin usually occur together in a lesion and suggest that it is aggressive. However, occasionally one may see a lesion with a narrow zone of transition but no sclerotic margin. This very unusual combination of determinants is found most commonly in giant cell tumor and less commonly in plasmacytoma. It is therefore useful to describe these determinants separately^16^.
8. Presence of visible tumor matrix (**Fig. 10**). The character of any tumor matrix should be described, since it may be tumor specific or may at least allow categorization of a lesion as bone producing versus cartilage producing. In general, aggressive bone-forming tumors produce amorphous osteoid, which is often less dense than normal bone. Less aggressive bone-forming tumors produce better organized, denser bone^17^. The matrix of cartilage- producing tumors is usually quite distinctive, appearing stippled and more dense than normal bone^18^.
9. Host response. An aggressive lesion may not allow a host response, demonstrating cortical destruction and penetration, with or without periosteal reaction. A less aggressive lesion may result in cortical thickening or sclerosis, cortical thinning without reactive bone formation, or cortical expansion^19^. It might be noted that the character of periosteal reaction is not always a reliable sign in determining the aggressiveness of the lesion. However, generally thin linear periosteal reaction is seen in less aggressive lesions while sunburst periosteal reaction is seen in the more aggressive categories^20^,^21^.
10. Polyostotic versus monostotic. This is the last determinant and might be the most important, since polyostotic lesions automatically restrict the number of disease processes that might be considered. For example, nonaggressive polyostotic lesions should be confined to fibrous dysplasia, Paget disease, histiocytosis, multiple exostosis, and multiple enchondromatosis^22^. Aggressive polyostotic lesions would be confined to osseous metastases, multiple myeloma, primary bone tumor with osseous metastases, an aggressive phase of Paget disease, multifocal osteomyelitis, aggressive histiocytosis, and multifocal vascular bone tumors^23^.

**Figure 1:**
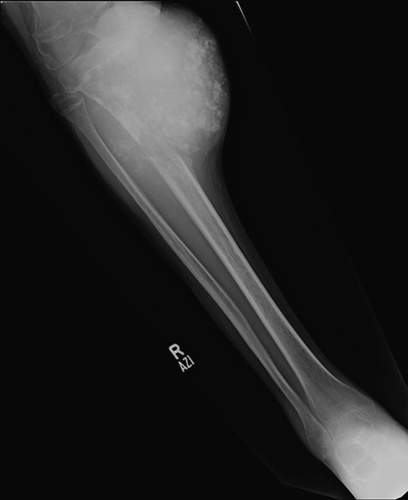
Cortical break with soft tissue component, an aggessive lesion.

**Figure 2:**
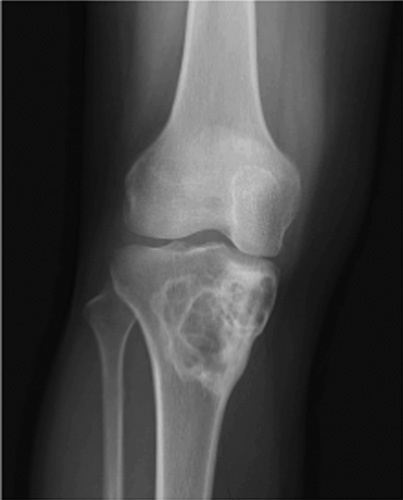
Eccentric geographic lesion with a sclerotic margin, a healing nonossifying fibroma is present in proximal tibia.

**Figure 3:**
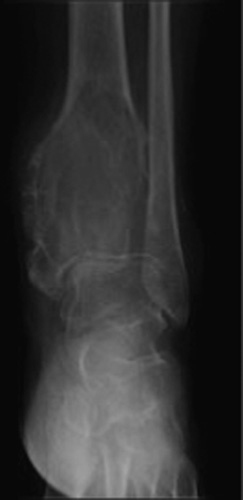
Geographic lesion with well-defined rim, usually a nonsclerotic margins. Giant cell tumor is seen involving the distal tibia. The lesion is expansile extending to the articular surface.

**Figure 4:**
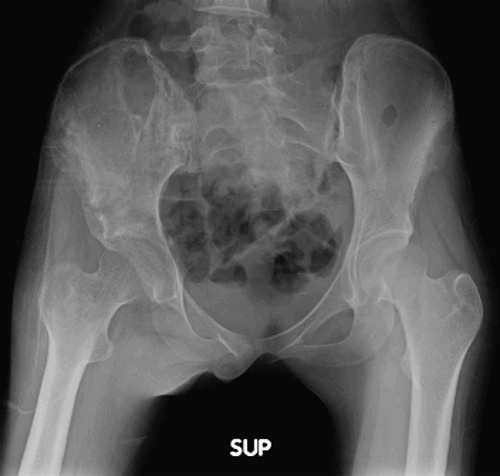
Ewing sarcoma with permeative lesion involving right iliac blade up to acetabulum.

**Figure 5:**
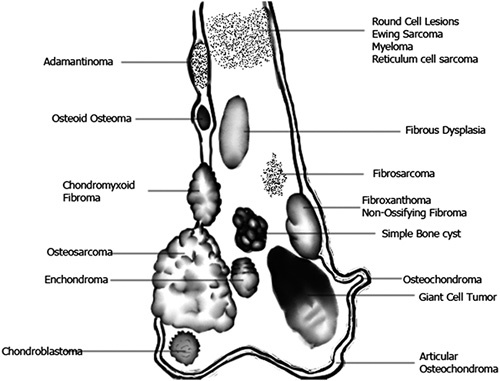
Location of common bone lesions.

**Figure 6:**
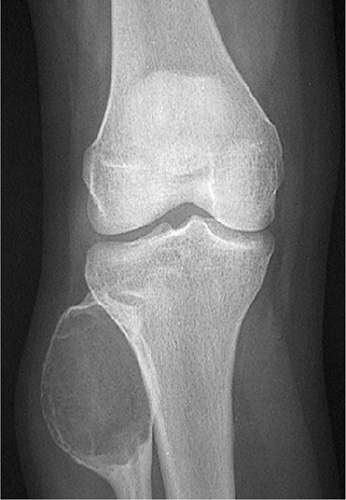
A lesion with narrow zone of transition.

**Figure 7:**
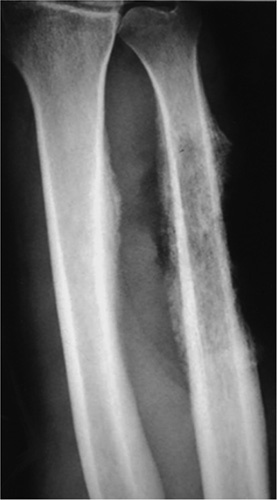
Lesion with wide zone of transition.

**Figure 8:**
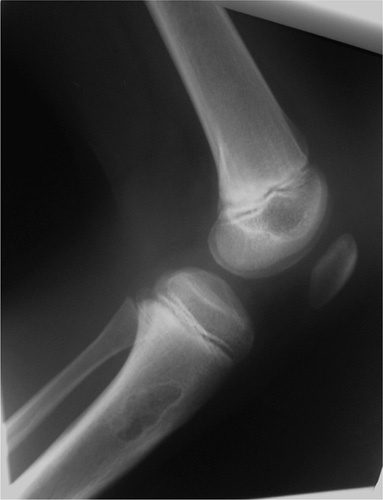
Bone lesion with sclerotic margins.

**Figure 9:**
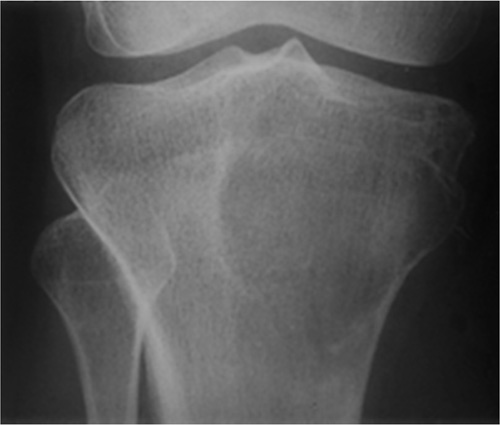
Bone lesion with nonsclerotic margins.

**Figure 10:**
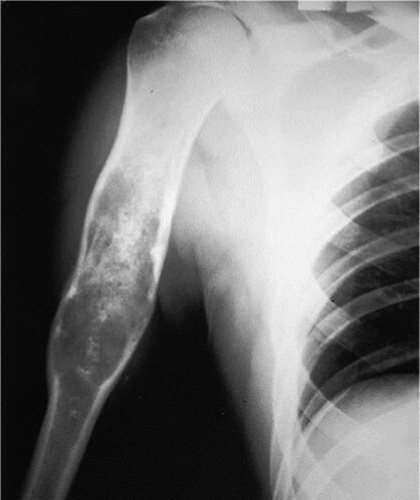
Bone lesion with a chondroid matrix, case of a chondrosarcoma.

The description of these 10 determinants should yield the diagnosis or at least a short differential diagnosis. The individual musculoskeletal tumors often have very characteristic features among these determinants. After several introductory cases, these sets of determinants will be discussed with each individual entity. If one cannot give a diagnosis, it is important to conclude with the observation of whether the lesion is aggressive or nonaggressive rather than malignant or benign. The reasoning here is that some malignant lesions may appear nonaggressive and several benign lesions often appear highly aggressive (especially osteomyelitis and histiocytosis)^24^. If one uses the term benign or malignant in one’s description, consideration of such lesions which may appear aggressive but act benign often will be precluded.

If the exact diagnosis is not reached after examination of the plain film, one might attempt to place the lesion in one of the following 5 categories:

1. An asymptomatic, benign leave-me-alone lesion, which requires no further imaging or attention. An example might be a fibrous cortical defect or classic nonossifying fibroma.
2. An asymptomatic, almost certainly benign lesion. Such a lesion could be safely followed without further workup and an example might be a large nonossifying fibroma.
3. A benign symptomatic lesion with a highly probable diagnosis with the region of involvement well seen. Examples of this might be a giant cell tumor or chondroblastoma. In many cases, one can proceed to definitive treatment without further imaging of these lesions.
4. A lesion of uncertain diagnosis and a mixture of aggressive and less aggressive features such that benign or malignant status cannot be confidently assessed. Although a good radiologist can attempt to keep the number of lesions assigned to this category small, some lesions truly belong to this category (such as a low-grade intermedullary chondrosarcoma or an aggressive giant cell tumor) and must be worked up carefully as if they truly belong to the most aggressive category.
5. An obviously malignant lesion which requires further workup, perhaps for diagnosis but certainly for staging.

–

**Figure:**
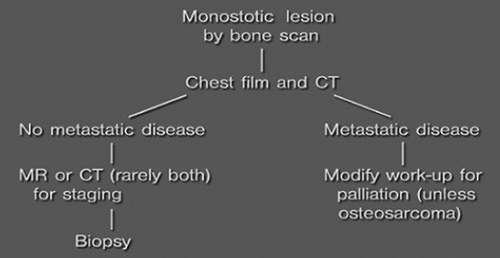


–

**Figure:**
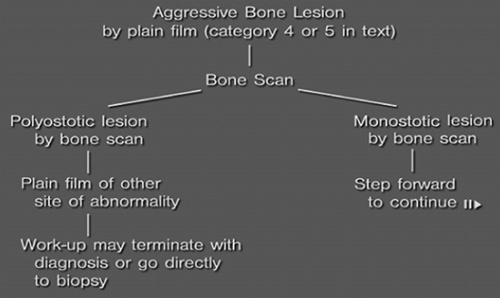


–

**Figure:**
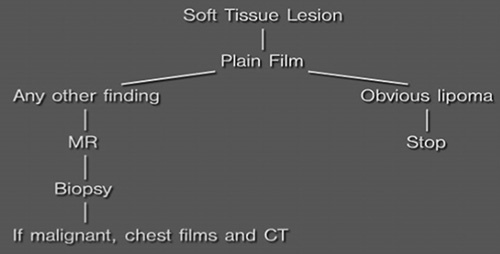


## Benign conditions with potential for malignant transformation^25^,^26^

**Table:**
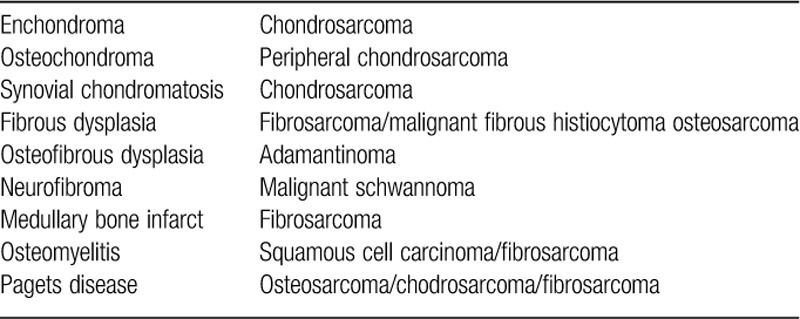


## Bone lesions usually lacking a sclerotic border^27^, ^28^,^29^

- Enchondroma in short tubular bones
- Giant cell tumor
- Brown tumor of hyper parathyroidism
- Osteolytic phase of pagets disease
- Acute osteomyelitis

## Bone lesions with usually a sclerotic border^30^,^31^

- Anuerysmal bone cyst
- Benign fibrous histiocytoma
- Bone abscess
- Chondroblastoma
- Chondromyxoid fibroma
- Epidermoid inclusion cyst
- Fibrous cortical defect
- Fibrous dysplasia
- Intraosseous ganglion
- Medullary bone infarct
- Nonossifying fibroma
- Osteoblastoma
- Osteofibrous dysplasia
- Periosteal chondroma
- Simple bone cyst

## Periosteal reaction

### Uninterrupted periosteal reaction^32^,^33^

**Table:**
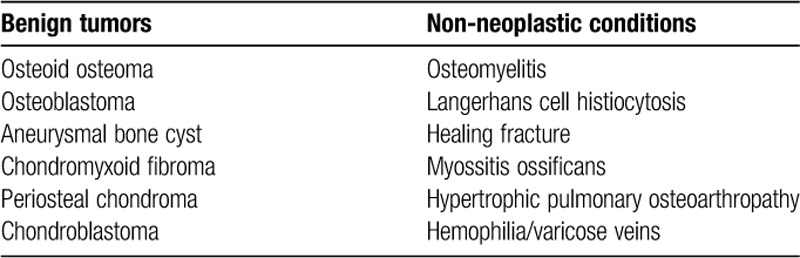


### Interrupted periosteal reaction

#### Non-neoplastic conditions^32^

- Osteomyelitis
- Langerhans cell histiocytosis
- Subperiosteal hemorrhage

## Benign lesions with aggressive features^30^,^31^

- Osteoblastoma (aggressive)
- Desmoplastic fibroma
- Periosteal desmoids
- Giant cell tumor
- Aneurysmal bone cyst
- Osteomyelitis
- Langerhans cells histiocytosis
- Pseudotumor hemophilia
- Myositis ossificans
- Brown tumor of hyperthyroidism

## Do not touch lesions

**Table:**
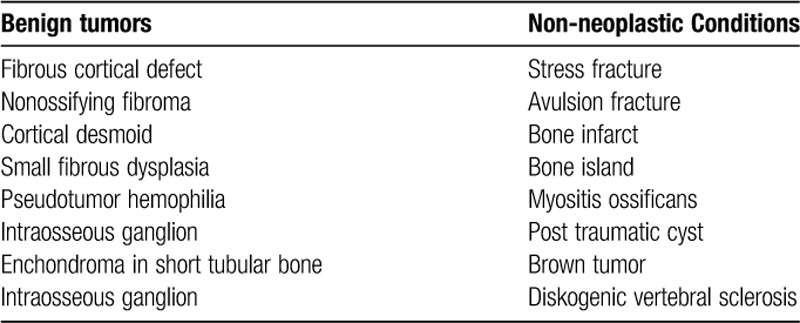


## Ethical approval

Exemption was obtained from Ethical Review Committee.

## Sources of funding

No funding from any organization.

## Author contribution

M.U. and O.H.A.H.: prepared the manuscript. D.K.: reviewed the radiological aspects of the manuscript and added the radiological graphs. N.U. and S.N.: revised the manuscript. All authors read and approved the final manuscript.

## Conflict of interest disclosures

The authors declare that they have no financial conflict of interest with regard to the content of this report.

## Research registration unique identifying number (UIN)

Not applicable.

## Guarantor

All authors read and approved the final manuscript and accept full responsibility for the work.

## References

[1] Biermann JS Common benign lesions of bone in children and adolescents. J Pediatr Orthop 2002;22:268–73. 11856945

[2] Sanders TG Parsons T Radiographic imaging of musculoskeletal neoplasia. Cancer Control 2001;8:221–31. 11378648 10.1177/107327480100800302

[3] Peterson JJ Current Developments and Recent Advances in Musculoskeletal Tumor Imaging Seminars in Musculoskeletal Radiology. Stuttgart; Germany: Thieme Medical Publishers; 2013. 10.1055/s-0033-1343093 23673541

[4] Plant J Cannon S Diagnostic work up and recognition of primary bone tumours: a review. EFORT Open Reviews 2016;1:247–53. 28461955 10.1302/2058-5241.1.000035 PMC5367572

[5] Kim S Lee S Arsenault DA Pediatric rib lesions: a 13-year experience. J Pediatr Surg 2008;43:1781–5. 18926207 10.1016/j.jpedsurg.2008.02.061

[6] Wyers MR Evaluation of pediatric bone lesions. Pediatr Radiol 2010;40:468–73. 20225104 10.1007/s00247-010-1547-4

[7] Dähnert W Radiology Review Manual. Philadelphia: Lippincott Williams & Wilkins; 2011.

[8] Qadir I Umer M Umer HM Managing soft tissue sarcomas in a developing country: are prognostic factors similar to those of developed world? World J Surg Oncol 2012;10:188. 22974324 10.1186/1477-7819-10-188 PMC3502247

[9] Ramachandran M Basic Orthopaedic Sciences: the Stanmore Guide. Boca Raton; Florida: CRC Press; 2006.

[10] Jain D Jain VK Vasishta RK Adamantinoma: a clinicopathological review and update. Diagn Pathol 2008;3:8. 18279517 10.1186/1746-1596-3-8 PMC2276480

[11] Budny AM Ismail A Osher L Chondromyxoid fibroma. J Foot Ankle Surg 2008;47:153–9. 18312923 10.1053/j.jfas.2007.08.013

[12] Sassoon AA Fitz-Gibbon PD Harmsen WS Enchondromas of the hand: factors affecting recurrence, healing, motion, and malignant transformation. J Hand Surg 2012;37:1229–34. 10.1016/j.jhsa.2012.03.019 22542061

[13] Zamora T Urrutia J Schweitzer D Do orthopaedic oncologists agree on the diagnosis and treatment of cartilage tumors of the appendicular skeleton? Clin Orthop Relat Res 2017;475:2176–86. 28205076 10.1007/s11999-017-5276-y PMC5539017

[14] Yildiz C Erler K Atesalp AS Benign bone tumors in children. Curr Opin Pediatr 2003;15:58–67. 12544273 10.1097/00008480-200302000-00010

[15] Skeletal Lesions Interobserver Correlation among Expert Diagnosticians (SLICED) Study Group. Reliability of histopathologic and radiologic grading of cartilaginous neoplasms in long bones. J Bone Joint Surg Am 2007;89:2113–2123. 17908885 10.2106/JBJS.F.01530

[16] Fletcher CD Unni KK Mertens F Pathology and Genetics of Tumours of Soft Tissue and Bone. Lyon; France: IARC; 2002.

[17] Lovell WW Winter RB Morrissy RT Lovell and Winter’s Pediatric Orthopaedics. Philadelphia: Lippincott Williams & Wilkins; 2005.

[18] Geirnaerdt MJ Hogendoorn PC Bloem JL Cartilaginous tumors: fast contrast-enhanced MR imaging 1. Radiology 2000;214:539–46. 10671608 10.1148/radiology.214.2.r00fe12539

[19] Yarmish G Klein MJ Landa J Imaging characteristics of primary osteosarcoma: nonconventional subtypes 1. Radiographics 2010;30:1653–72. 21071381 10.1148/rg.306105524

[20] Murphey MD Robbin MR MCRae GA The many faces of osteosarcoma. Radiographics 1997;17:1205–31. 9308111 10.1148/radiographics.17.5.9308111

[21] Greenspan A Orthopedic Imaging: A Practical Approach. Philadelphia: Lippincott Williams & Wilkins; 2011.

[22] Cronin MV Hughes TH Bone tumors and tumor-like conditions of bone. Appl Radiol 2012;41:6.

[23] Meyers SP MRI of Bone and Soft Tissue Tumors and Tumorlike Lesions. Stuttgart; Germany: Thieme New York, NY; 2008.

[24] Halperin EC Constine LS Tarbell NJ Pediatric Radiation Oncology. Philadelphia: Lippincott Williams & Wilkins; 2012.

[25] Horvai A Unni KK Premalignant conditions of bone. J Orthop Sci 2006;11:412–23. 16897210 10.1007/s00776-006-1037-6 PMC2780648

[26] Kansagra A Wan J Devulapalli K Malignant transformation of an aneurysmal bone cyst to fibroblastic osteosarcoma. Am J Orthop 2016;45:E367–E72. 27737291

[27] Manzil FFP Bhambhvani P Vattoth S Primary hyperparathyroidism-related brown tumors mimicking other giant cell–containing skeletal tumors: role of correlative imaging in diagnosis. J Nucl Med Technol 2013;41:46–8. 23385541 10.2967/jnmt.112.115204

[28] Mohan M Neelakandan RS Siddharth D An unusual case of brown tumor of hyperparathyroidism associated with ectopic parathyroid adenoma. Eur J Dent 2013;7:500. 24932128 10.4103/1305-7456.120657 PMC4053678

[29] Remotti F Feldman F Nonneoplastic lesions that simulate primary tumors of bone. Arch Pathol Lab Med 2012;136:772–88. 22742550 10.5858/arpa.2011-0557-RA

[30] Girish G Finlay K Morag Y Imaging review of skeletal tumors of the pelvis—part I: benign tumors of the pelvis. Scientific World Journal 2012;2012:290930. 22666102 10.1100/2012/290930 PMC3362015

[31] Helms CA Fundamentals of Skeletal Radiology. Philadelphia: Saunders; 2004.

[32] Zaveri J La Q Yarmish G More than just Langerhans cell histiocytosis: a radiologic review of histiocytic disorders. Radiographics 2014;34:2008–24. 25384298 10.1148/rg.347130132

[33] Costelloe CM Madewell JE Radiography in the initial diagnosis of primary bone tumors. Am J Roentgenol 2013;200:3–7. 23255735 10.2214/AJR.12.8488

